# Systematic and detailed analysis of behavioural tests in the rat middle cerebral artery occlusion model of stroke: Tests for long-term assessment

**DOI:** 10.1177/0271678X16654921

**Published:** 2016-01-01

**Authors:** Rebecca C Trueman, Claris Diaz, Tracy D Farr, David J Harrison, Anna Fuller, Paweł F Tokarczuk, Andrew J Stewart, Stephen J Paisey, Stephen B Dunnett

**Affiliations:** 1 School of Life Sciences, University of Nottingham, Nottingham, UK; 2 Brain Repair Group, School of Biosciences, Cardiff University, Cardiff, UK; 3 Institute of Infection and Immunity, School of Medicine, Cardiff University, Cardiff, UK; 4 Imaging Sciences Department, MRC Clinical Sciences Centre, Faculty of Medicine, Imperial College, UK; 5 EMRIC, Biosciences, Cardiff University, Cardiff, UK; 6 PETIC, School of Medicine, Cardiff University, Cardiff, UK

**Keywords:** Animal models, behaviour (rodent), brain recovery, focal ischemia, immunohistochemistry, stroke, MCAO

## Abstract

In order to test therapeutics, functional assessments are required. In pre-clinical stroke research, there is little consensus regarding the most appropriate behavioural tasks to assess deficits, especially when testing over extended times in milder models with short occlusion times and small lesion volumes. In this study, we comprehensively assessed 16 different behavioural tests, with the aim of identifying those that show robust, reliable and stable deficits for up to two months. These tasks are regularly used in stroke research, as well as being useful for examining striatal dysfunction in models of Huntington’s and Parkinson’s disease. Two cohorts of male Wistar rats underwent the intraluminal filament model of middle cerebral artery occlusion (30 min) and were imaged 24 h later. This resulted in primarily subcortical infarcts, with a small amount of cortical damage. Animals were tested, along with sham and naïve groups at 24 h, seven days, and one and two months. Following behavioural testing, brains were processed and striatal neuronal counts were performed alongside measurements of total brain and white matter atrophy. The staircase, adjusting steps, rotarod and apomorphine-induced rotations were the most reliable for assessing long-term deficits in the 30 min transient middle cerebral artery occlusion model of stroke.

## Introduction

Many rodent models of focal brain ischemia exist, but the most commonly employed is the intraluminal filament middle cerebral artery occlusion (MCAO) model of stroke.^1^ There is debate regarding the ability of different behavioural tasks to reveal reliable deficits in this model. Often rats recover sensorimotor function in the weeks following MCAO; many groups have shown that MCAO rats exhibit recovery or even compensation, particularly if tested for more than seven days.^2^ Despite this, few studies assess behaviour for more than a few days post-ischemia. The Stroke Therapy Academic Industry Roundtable (STAIR) Preclinical Recommendations highlighted that measurement of behavioural outcomes for testing novel therapeutics should occur for at least two to three weeks post induction of focal ischemia.^3^ This is particularly important when considering the plethora of pathological changes that are occurring in the brain following the initial event. If chronic time points are not included, researchers cannot be certain that any potential intervention will have a long-term functional effect, which will be required for any novel therapy to progress to testing in patients. Functional outcomes are often not assessed until three to six months post-stroke in clinical trials and, as in the rodent, patients often display a degree of recovery in the first few weeks following the stroke. Thus, to be able to develop therapeutics which will translate from bench to bedside, it is necessary to find stable long-term tests of neurological function in animal models. These tests need to be easy to administer, and preferably quantitative rather than qualitative in nature to reduce the risk of bias.

In the current study, we comprehensively assessed the MCAO filament model of stroke in rats using 16 different behavioural tests. We evaluated tests that are commonly used in stroke models and introduce other tests that have proven extremely successful for detailed assessment of striatal dysfunction in models of Parkinson’s and Huntington’s disease. The aim was to identify tests that are sensitive to MCAO, and which show robust, reliable and stable deficits for up to two months. For this purpose, we chose a relatively specific lesion model with a small infarct volume, which would be more suitable for longer term studies of neuroprotective and neurorepair strategies.

## Materials and methods

A brief outline of the methods is below. Full details are provided in the supplemental methods.

### Animals

Seventy-five male Wistar rats were divided into two cohorts (10–12 weeks old; Harlan, UK) and housed in groups of three to four, in standard cages with wood shavings as bedding, nesting material, a tube and aspen chew blocks. Prior to surgery, stratified randomisation was performed and animals were assigned to sham, naïve or MCAO groups based on baseline behaviour scores to negate any potential pre-existing differences in the groups. Following surgery animals were kept in the same cage groups where possible, meaning sham, naïve or MCAO animals were often housed together. The temperature (21 ± 2℃), humidity (55 ± 10%) and light cycle (14/10 hour light/dark cycle) of the environment was controlled. All experiments were performed within the same facility in accordance with the UK Animals (Scientific Procedures) Act 1986, under a project license approved and granted by the UK Home Office. Experiments are reported in accordance with the ARRIVE guidelines.

Each experiment utilised a pre-defined exclusion criteria: (i) no restitution of cerebral blood flow (CBF) following removal of the filament (indicative of haemorrhage), n = 15 (all from the MCAO group); (ii) incomplete MCAO, defined as absence of observable cortical or striatal changes during MRI at 24 h, n = 11 (all from the MCAO group); and (iii) post-operative weight loss of more than 20% of pre-surgical weight, n = 2 (all from the MCAO group). Additionally, one sham animal showed abnormalities at 24 h in the MRI and two other MCAO animals had complications during surgery. All of these animals were excluded prior to post-surgical behavioural testing.

The remaining 44 animals were allocated as follows: Cohort 1: 11 MCAO, 8 sham and 6 naïve animals; Cohort 2: 9 MCAO, 5 sham and 5 naïve controls. The sham and naïve controls were subsequently combined to make one control group in each cohort following comparison to ascertain the absence of differences between them (supplemental methods A5). Sample sizes were selected based on previous work with other models of striatal injury on a selection of tasks, and with the rational that if deficits were not robust with these group sizes then studies for treatments, which would require significantly larger groups, would not be feasible.

### Middle cerebral artery occlusion

Sham and 30 min MCAO procedures were carried out under isoflurane anaesthesia, using the method described in Trueman et al.^4^ to prevent damage to the facial musculature, as described fully in the supplemental methods (A1). During the procedure, blood flow was monitored using a Laser Doppler Perfusion Monitor (Moor Instruments, Axminster, UK).

### MRI measurements and image analysis

Twenty-four hours following surgery, sham and MCAO animals underwent a structural Multi-Slice Multi-Echo (MSME) T_2_-weighted MRI scan using a 9.4TBiospec with a rat head surface coil (Bruker BioSpin, Ettlingen, Germany). Analyze software (AnalyzeDirect, Overland Park, USA) was used to delineate manually the infarct and T_2_ values (ms) were recorded, as described fully in the supplemental methods (A2).

### Behavioural testing

Methods and full protocols are provided for each behavioural test in the online supplemental data (Sections A and C), including practical advice and troubleshooting tips (C). All behavioural testing was performed by a blinded observer. Two cohorts of rats were used to reduce the number of tests each rat was exposed to.

### Food restriction

Cohort 1 required food restriction to perform the food reward tasks. Their body weight was maintained at 85–90% of free feeding weight, by giving restricted amounts of food daily. This commenced one week prior to baseline training and rats were returned to ad libitum feeding a week prior to surgery until three days prior to the commencement of testing at four weeks post-MCAO.

### Experimental design

**Figure 1.**
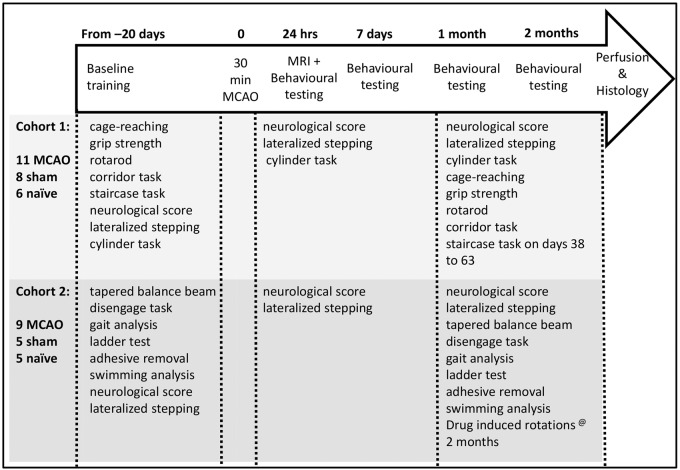
Outline of the experimental design and tasks performed by Cohort 1 and 2.

All rats were trained on the behavioural tasks prior to surgery and were tested at one and seven days, and one (4–5 weeks) and two months (8–9 weeks) post-MCAO (Figure 1). Behavioural testing was carried out between the hours of 8 a.m. and 7 p.m. and animals were tested in cage order.

Following MCAO, both cohorts were tested for less than 12 min a day, with an extra 15 min a day for 25 days when Cohort 1 was tested on the staircase task, and an extra 150 min of drug-induced rotations (apomorphine & amphetamine) at the end of the study for Cohort 2.

### Tissue processing and immunohistochemistry

As described fully in the supplemental methods (A4), following perfusion fixation brains were sectioned and stained for NeuN (1:4000, Chemicon, UK) and solochrome cyanine (Sigma Aldrich, UK^5^) to allow visualization of neuronal nuclei and white matter tracts, respectively. Sections were digitally photographed to determine final tissue and white matter loss using Image J (NIH, USA).

Atrophy was calculated as

(1) Wholebrainatrophy=100×((Hc-Vc)-(Hi-Vi))/(Hc-Vc)

(2) Whitematteratrophy=100×(Wc-Wi)/Wc

where H, V, W, *i*, and *c* represent the volumes of the contralateral (*c*) and ipsilateral (*i*) hemisphere (H), ventricle (V), and white matter-stained (W) areas.

Additionally, stereological neuronal counts of the entire right striatum of a 1 in 12 series using the Olympus C.A.S.T. grid system v1.6 were performed to estimate the total neuronal count for the striatum. One MCAO and one control animal from Cohort 1 and one control animal from Cohort 2 were excluded from the histological analysis due to tissue damage.

### Statistical analysis

For full details of statistical analysis, see the supplemental methods (A5). Briefly, behavioural data were tested for normality, and those measures which were normally distributed (rotarod, balance beam (latency to cross), corridor and gripstrength (force)) were analysed using a repeated measures ANCOVA, with baseline performance used as a co-variate. Those measures that were not normally distributed were analysed using the non-parametric Mann–Whitney test. Simple comparisons for normally distributed histological data were analysed using Student’s *t*-tests.

## Results

### Neuropathology

**Figure 2.**
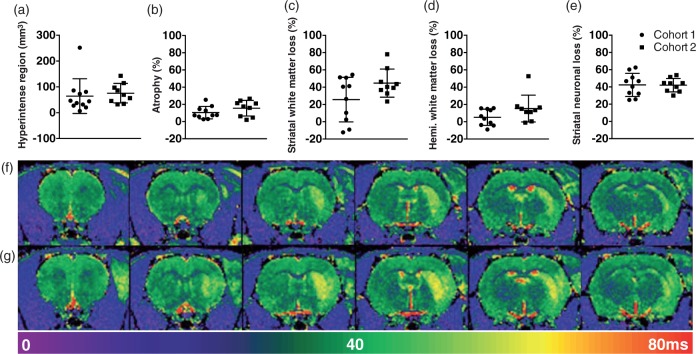
Assessment of MCAO neuropathology in Cohorts 1 and 2. (a) Hyperintense volume on MRI at 24 h post MCAO surgery. (b) Percentage of hemispheric atrophy from histology at two months. (c) Percentage of striatal white matter atrophy, and (d) Percentage of total hemisphere white matter atrophy calculated from solochrome cyanine stained sections at two months. (e) Striatal NeuN cell loss as a percentage of the total number of cells in the Control group. (f) and (g) Representative T_2_ maps from animals in Cohorts 1 and 2, respectively. All graphs show mean ± standard deviation (SD), n = 11 and 9 for MRI and 10 and 9 for all other measures. No signifcant differences were found between the two Cohorts.

The lesions-induced cell loss in the lateral neocortex and striatum on the side of the infarction and atrophy of the hemisphere. The T_2_ maps indicated greater variability in the extent of the hyperintensity in Cohort 1 (Figure 2(f)) than Cohort 2 (Figure 2(g)), although this was not significant (Figure 2(a), U = 31, n.s.). Similarly, the MCAO-lesioned animals in the two Cohorts did not exhibit significantly different infarct volumes (i.e. hemispheric atrophy), striatal or hemispheric white matter loss, or striatal neuronal loss (Figure 2(b) to (e); t_17_ = 1.37, t_17_ = 1.90, U = 25 and t_17_ = 0.05, respectively, all n.s.).

### Behavioural measures

For clarity of presentation, we have grouped the tests into four different broad categories: tests in which robust long-term deficits were observed in MCAO animals, tests in which the impairment was subtle and/or recovered spontaneously, tests in which the results were counter-intuitive, and tests in which no significant impairments in MCAO animals were observed.

#### Tests that uncovered significant and stable deficits in the MCAO animals

##### Staircase task

**Figure 3.**
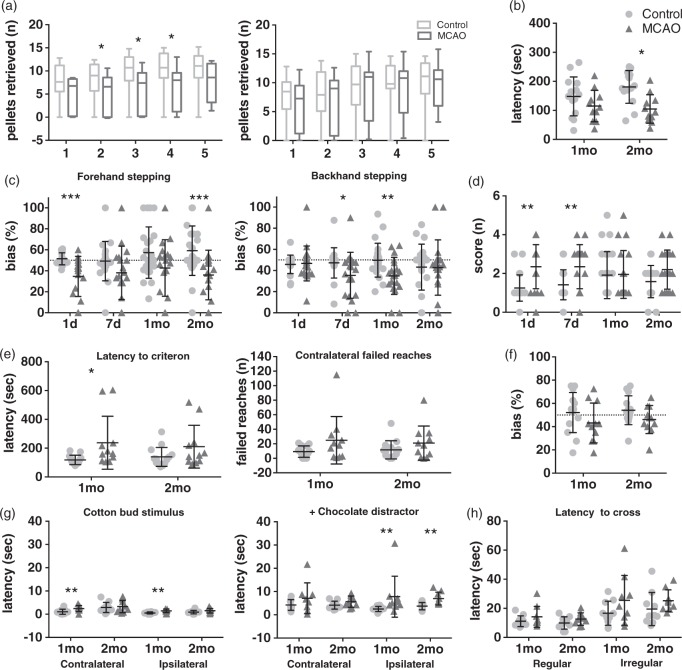
Summary of tests in which MCAO animals exhibited deficits. (a) Pellets retrieved with each forelimb in the staircase task at two months (Cohort 1: n = 14 Control, n = 11 MCAO). (b) Latency to fall from the Rotarod at one and two months (Cohort 1: n = 14 Control, n = 11 MCAO). (c) Forehand and backhand steps during lateralized stepping (both cohorts at one day: n = 19 Control, n = 20 MCAO, at seven days: n = 19 Control, n = 19 MCAO, at one month: n = 23 Control, n = 18 MCAO, and at two months: n = 24 Control, n = 20 MCAO). (d) Modified neuroscore (both cohorts: n = 24 Control, n = 20 MCAO). (e) Time to criterion and number of failed reaches in the cage reaching task at one and two months (Cohort 1: at 1 mo: n = 13 Control, n = 11 MCAO, at two months: n = 13 Control, n = 11 MCAO). (f) Bias in the corridor task at one and two months (Cohort 1: n = 14 Control, n = 11 MCAO). (g) Latency to respond with and without the chocolate distractor in the disengage task (Cohort 2: n = 10 Control, n = 9 MCAO, except at one month with chocolate distractor for the contralateral paw: n = 10 Control, n = 8 MCAO). (h) Latency to cross for regular and irregular rungs in the ladder test at one and two months (Cohort 2: n = 10 Control, n = 9 MCAO). Data are expressed as median, minimum and maximum values in the box and whisker plots and as means ± standard deviation (SD) with individual values in the scatter plots. Note: the dotted line represents 50% bias (no preference for either side or paw). * = *p* < 0.05, ** = *p* < 0.01, *** *p* < 0.001.

MCAO rats collected fewer pellets with their contralateral paw, than the corresponding paw in the Control group, on the staircase test at two months post-surgery (Figure 3(a): Blocks 2–4: U = 38–41, *p* < 0.05, Block 5: U = 45, *p* = 0.08), whereas they showed no deficits in the number of pellets retrieved on the ipsilateral side (U = 59–74, n.s.).

##### Apomorphine-induced rotation

The MCAO animals exhibited a significant increase in the number of rotations to the ipsilateral side in response to apomorphine; this was in contrast to no net asymmetry in rotations in the Control animals (MCAO, 189.0 ± 169.3 vs. Controls, 3.4 ± 9.23 (Mean ± SD); U = 14, *p* < 0.01).

#### Tests in which the MCAO animals exhibited subtle deficits and/or recovery

##### Rotarod

Latency to fall off the Rotarod was significantly lower in the MCAO group when compared to the Control (Figure 3(b), F_1,22_ = 6.59, *p* < 0.05). This deficit appeared to increase over time (MCAO × Time: F_1,23_ = 8.95, *p* < 0.01) such that the post hoc comparison between groups was only significant on the two-month time point (*p* < 0.05). However, this effect was not due to a further decrease in performance in the MCAO group, but rather an improvement in the ability to remain on the Rotarod in the Control group.

##### Lateralized stepping

The MCAO rats exhibited a paw bias, meaning they performed fewer steps with the contralateral paw than with the ipsilateral paw, although this deficit was not stable across all time points. They had a paw bias in the forehand direction at one day and two months (Figure 3(c), U = 72, *p* < 0.001, U = 100.5, *p* < 0.001) and in the backhand direction at seven days and one month (Figure 3(c), U = 105, *p* < 0.05, U = 114, *p* < 0.01).

##### Modified neuroscore

The score differed between MCAO and Control groups at one and seven days post-MCAO (Figure 3(d); U = 104.0 and 123.5, respectively, both *p* < 0.01); at both time points, the MCAO animals scored higher (indicating reduced neurological function overall) but this was no longer the case at one and two months (U = 212.5 and 162.0, respectively, both n.s.).

##### Cage reaching task

MCAO rats took longer to achieve 20 successful reaches than Control animals at one month (Figure 3(e), U = 28, *p* < 0.05). However, no other measures achieved significance.

##### Corridor task

MCAO animals retrieved fewer pellets from the contralateral side in the corridor task at both one and two months after surgery, in comparison to the absence of a side preference in the Control group. However, this difference did not achieve significance (Figure 3(f), Groups, F_1,22_ = 4.14, *p* = 0.054).

##### Disengage task

Overall a larger response time to both contralateral and ipsilateral perioral stimulation without the chocolate distraction was evident in the MCAO group in comparison to the Control group at one month (Figure 3(g), Contra: U = 15.50, *p* < 0.05, Ipsi: U = 13, *p* < 0.01), but not at two months (U = 43, 29, n.s.). When the chocolate distraction was introduced, there was a significantly higher response time to perioral stimulation in the MCAO group on the ipsilateral side in comparison to the Control group at one month (U = 11, *p* < 0.01) and two months (U = 10, *p* < 0.01).

##### Ladder test

On the regular spaced rungs in the ladder task, there was a small but significant increase in the number of hindpaw slips exhibited by the MCAO group at one month (Control 0.0 ± 0.0, MCAO 0.08 ± 0.08 (Mean ± SD); U = 20, *p* < 0.05). However, there was also a reduction in forepaw slips in the MCAO group at one month (Control 0.50 ± 0.40, MCAO 0.20 ± 0.36 (Mean ± SD); U = 20.5, *p* < 0.05). It should be noted that the number of overall foot slips made on the ladder with regular rungs was very low (means of 0.1–0.5 slips per traverse) and both groups crossed relatively quickly (10–12 s; Figure 3(h)). In comparison, rats took almost twice as long to cross the ladder with irregularly spaced rungs (Figure 3(h), 18–25 s). This slowing was most apparent in the MCAO group, but failed to achieve significance (U = 29 & 24, n.s.).

#### Tests in which the results were counter-intuitive.

##### Swimming analysis

**Figure 4.**
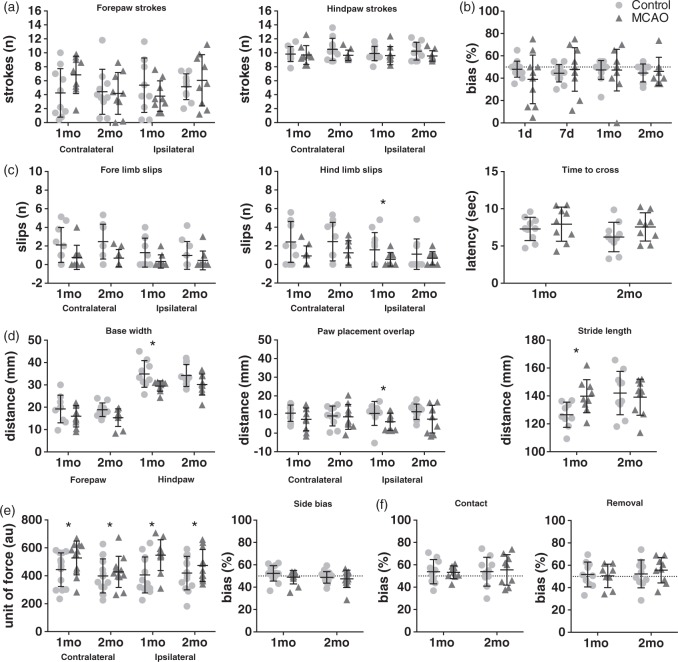
Summary of tests in which the MCAO animals exhibited no deficits (b and f) or tests that were counter-intuitive (a, c, d, and e). (a) Number of fore and hindpaw strokes during swimming analysis at one and two months (Cohort 2: n = 10 Control, n = 9 MCAO). (b) Forelimb exploring bias in the cylinder task (Cohort 1: at one day: n = 14 Control, n = 11 MCAO, at seven days: n = 12 Control, n = 11 MCAO, at one month: n = 14 Control, n = 11 MCAO, and at two months n = 10 Control, n = 8 MCAO). (c) Hind and forepaw slips and time to cross the tapered balance beam at one and two months(Cohort 2: n = 10 Control, n = 9 MCAO). (d) Paw base width, paw placement overlap, and stride length during gait analysis at one and two months (Cohort 2: at one month: n = 9 control, n = 9 MCAO, and at two months: n = 10 Control, n = 9 MCAO). (e) Force required to release and side bias during grip strength at one and two months(Cohort 1: n = 14 Control, n = 11 MCAO). (f) Contact and removal bias during adhesive removal at one and two months(Cohort 2: n = 10 Control, n = 9 MCAO, except at one month for remove: n = 9 Control, n = 9 MCAO). Data are expressed as means ± SD with individual values in the scatter plots. Note: The dotted line represents 50% bias (no preference for either side or paw). * = *p* < 0.05, ** = *p* < 0.01, *** *p* < 0.001.

There were no significant differences in the number of fore or hindlimb strokes performed by the MCAO animals on either side when compared to the corresponding paws from the Control group (Figure 4(a), U = 24–44, n.s.). However, the MCAO animals were significantly faster at crossing the swim tank than the control animals (1 month: 2.26 ± 0.37 vs 2.82 ± 1.77 s, U = 10, *p* < 0.01. 2 month: 2.41 ± 0.34 vs 3.03 ± 0.81 s, U = 14, *p* < 0.01 (Mean ± SD))

##### Tapered balance beam

The MCAO group made significantly fewer contralateral forelimb slips on the beam in comparison with the control group at two months (Figure 4(c); U = 20.5, *p* < 0.05). However, MCAO animals were no faster in their latencies to cross the beam (F_1,15_ = 3.57, n.s).

##### Gait analysis

A number of measures of gait were assessed. Distance between forepaws (base width) was unaltered in the MCAO group compared to the Control group (Figure 4(d); n.s.). Hindlimb base width (distance between the two hind limbs) was narrower in the MCAO group than the Control rats at one month only (Hind base width, Figure 4(d): U = 14, *p* < 0.05). Overlap of hind and forepaws during the walking cycle appeared to be reduced in the MCAO animals in comparison to the Control rats; however, this only achieved significance on the ipsilateral side at one month (U = 18, *p* < 0.05). Additionally, stride length was only significantly increased in the MCAO group in comparison to the Control group at the one-month time point (Figure 4(d); U = 13.5, *p* < 0.05).

##### Grip strength

The MCAO group required significantly more force than control animals to release the bars at both one and two months (Figure 4(e); Groups; F_1,22_ = 4.36, *p* < 0.05). Moreover, they exhibited no significant asymmetry between the two grip strength bars (Bias: n.s.) indicating no difference in grip strength between sides.

#### Tests in which the MCAO animals did not exhibit significant deficits.

##### Amphetamine-induced rotations

Neither group exhibited any rotational asymmetries in response to amphetamine (net rotations: Control: 7.10 ± 46.75 vs. MCAO: −8.22 ± 121.2, (Mean ± SD); n.s.).

##### Adhesive removal

The adhesive removal (‘sticky label’) test of somatosensory neglect showed no differences between groups at either time point (one or two months) in either the latency to contact or remove the labels affixed to either of their forepaws (Figure 4(f)).

##### Cylinder task

Although there appeared to be a small contralateral deficit in paw placing on the surface of the cylinder among the MCAO animals at one day, this was not significant and no significant bias in forelimb exploratory use was observed at any of the other post-surgical time points (Figure 4(b))

#### Correlations of behavioural data with histological/MRI measures

A multiple correlational analysis was undertaken between the most robust tests (staircase task and apomorphine rotations), those that would be of utility (rotarod and lateralized stepping), and the five measures of neuropathology (MRI hyperintensity; hemispheric, total white matter and striatal white matter atrophy; and striatal neuronal number). Individual results are summarised in Supplementary Table S1. Apomorphine-induced rotations, rotarod and forehand lateralised stepping did not correlate with histological measures. In the staircase reaching test, the number of pellets retrieved on the contralateral side over the last five days of training correlated positively with the number of surviving striatal neurons (ρ = 0.8, *p* < 0.01), and the hyperintense region on the MRI (ρ = −0.63, *p* < 0.05). Additionally, the hyperintense region of the MRI significantly correlated will all neuropathological measures apart from the number of ipsilateral striatal neurons (ρ = 0.58–.72, *p* all < 0.01).

### Discussion

This extensive comparison of the most common tests for assessing motor and sensorimotor function following MCAO in rats has provided some interesting results. We consider that the most useful way of summarizing the tests is to rank them in terms of utility according to the underlying aim, which was to identify tests that are suitable at long-term time points in order to monitor stable deficits and the effects of potential therapeutics. Therefore, the tests were split into four categories: those which uncovered robust long-term deficits for up to two months in MCAO animals, those in which MCAO animals exhibited subtle impairments but were not stable across time, those which failed to detect deficits in MCAO animals, and those in which the results appeared to be counter-intuitive, but still of potential utility. For a summary of this see Table 1. In order to further inform on the usefulness of these tests and assist in the design of future experiments, we performed sample size calculations on our data to compute group sizes required in order to observe a deficit in MCAO animals when compared to Controls at two months for tests/measures that were not significant in this study (Table 2). This was taken a step further for the tests we deemed most useful, and sample size calculations were performed on the MCAO data compared to a hypothetical MCAO group in which a 50% treatment effect would be required (Table 3).

**Table 1.** Summary of results. The tests in bold detected significant differences between the control and MCAO groups.

	1 day	7 days	1 month	2 months
Both cohorts	**Modified neurological score lateralized stepping**	**Modified neurological score lateralized stepping**	Modified neurological score **lateralized stepping**	Modified neurological score **lateralized stepping**
Cohort 1	Cylinder task	Cylinder task	Cylinder task **Cage reaching Grip strength** Rotarod Corridor task	Cylinder task Cage reaching **Grip strength Rotarod** Corridor task staircase task* (*on days 38 to 63)
Cohort 2			Tapered balance beam **Disengage task Gait analysis Ladder test** Adhesive removal **Swimming analysis**	**Tapered balance beam Disengage task** Gait analysis Ladder test Adhesive removal **Swimming analysis** Drug-induced rotations: **Apomorphine,** Amphetamine

**Table 2.** Sample size calculations for MCAO versus Control, based on the variance and difference between groups at the 2 month time point. Power = 0.8, α = 0.05.

Test	Measure	Sample size (per group)
Gait analysis	Hind paw base width	24
Disengage with distractor	Latency (contralateral)	31
Tapered balance beam	Latency	33
Modified neuroscore		36
Cage reaching	Latency to criterion	37
Corridor	Bias	38
Irregular ladder	Latency	44
Cage reaching	Failed reaches (contralateral)	59
Adhesive removal	Bias (remove)	208
Drug-induced rotations	Amphetamine	473
Disengage without distractor	Latency (contralateral)	552
Cylinder	Bias	818
Adhesive removal	Bias (contact)	1137
Regular ladder	Hind foot slips	10748

**Table 3.** Sample size calculations for MCAO versus treated MCAO (with anticipated 50% in improvement) based on the variance and difference between groups at the 2 month time point. Power = 0.8, α = 0.05.

Test	Measure	Sample size (per group)
Swimming analysis	Latency	20
Rotarod	Latency	27
Drug-induced rotations	Apomorphine	50
Lateralised stepping	Bias (forehand)	66
Staircase reaching	Pellets retrieved (contralateral)	84
Gait analysis	Hindpaw base width	87
Irregular ladder	Latency	107
Tapered balance beam	Latency	124
Corridor	Bias	145
Disengage with distractor	Latency (contralateral)	168
Modified neuroscore		168
Cage reaching	Latency to criterion	277
Gripstrength	(AU)	279
Cage reaching	Failed reaches (contralateral)	394

### Robust tests

Apomorphine-induced rotations robustly detected rotational bias in the MCAO animals at two months, which is consistent with other reports.^6^ The variability of this test was low, and as such achievable group sizes (n = 50, Table 3) would be required in order to test a therapeutic intervention. However, the interaction between apomorphine with any therapy should be carefully considered. The other test that proved to be useful at long-term time points was skilled reaching using the staircase chambers. This is not entirely surprising as skilled reaching requires a high degree of motor coordination and tests like this have been consistently used by several groups to reveal subtle impairments and compensatory behaviours in many different animal models of neurological disease and degeneration.^7^ Unfortunately, tests such as these are not routinely used in pre-clinical stroke research. This might be due to the fact that pre-training the animals to perform in the test apparatus can be quite labour intensive and may extend over several weeks in order to first achieve stable baseline performance. Furthermore, there is the additional requirement of food restriction in order to motivate the animals to perform. This may conflict with the ability of this test to be employed in the acute MCAO post-operative period and may render the tests undesirable for many groups. Furthermore, as food restriction has been shown to induce long-term alterations in rodents, which may be beneficial, such as improving behavioural Furthermore, enhancing neuroprotection and increasing longevity.^8,9^ As is the case with the majority of behavioural assessments, there is variability between animals, so large group sizes are often required and this can be even more discouraging with tests that require large investment such as skilled reaching. Within our study, the staircase deficit was stable across blocks 2–4 of training, and approached significance on block 5. Therefore, it would be recommended to increase the sample size for this test, indeed large group sizes would be required for testing treatments (Table 3). A second type of skilled reaching, caged skilled reaching, did not reveal stable deficits. This may have been due to the variability of the data and the propensity of the rodents to compensate by using the non-affected paw (a factor that is not contributing in the staircase task where pellets on each side of the body can only be retrieved with the corresponding paw). As well as increasing the group size to account for this variability (n = 37, Table 2), some groups lesion the side of the brain that corresponds to the preferred paw of each individual rodent.

### Tests in which MCAO animals exhibited subtle or transient impairments

A number of tests showed impairments at some time points, and therefore could potentially be of utility with increased groups sizes or additional training. The rotarod has been used for detecting deficits in the MCAO animals by many groups,^10,11^ and the data presented here supports this. At one month, the MCAO group was just shy of being significantly different from the Controls, though a significant difference was evident at two months. Care in interpretation of these data is crucial: performance was stable in the MCAO group at both time points, whereas performance improved in the Control animals, indicating a significant degree of motor learning in the latter group.^12^ Therefore, it may be advantageous to expose the animals to extra training until stable/asymptotic performance is achieved both prior to and following surgery. Based on the two-month data, a sample size of only 27 (Table 3) would be required to detect a treatment effect. The corridor task, which is highly sensitive in rodent models of Parkinson’s disease,^13^ appeared to be somewhat useful for assessing deficits in the MCAO group as there was a slight bias that only just failed to achieve significance. Indeed, we have repeated this test in other groups of MCAO rats with 60 min occlusion and found that it is discriminatory between MCAO and Control rats. Based on the current data, sample size calculations indicate that group sizes of 38 animals would be required to observe deficits between Control and MCAO rats.

Lateralized stepping was not consistent across all time points. Making adjusting steps in the forehand direction proved to be most difficult for MCAO rats at one day and two months: the same has been shown for the forelimb direction after distal MCAO (that affects only the cortex) out to eight weeks.^14^ Backhand adjustment steps were most difficult at seven days and one month. Different measures from this task did reveal deficits across all time points, and as such, it should not be ruled out, particularly since this test is simple to administer.^15^ The disengage task has been shown to reveal robust deficits in other animal models of striatal or nigro-striatal injury.^16^ The ability to ‘disengage’ from feeding when exposed to a sensory stimulus (cotton bud stimulating the perioral region) on either side (impaired or intact) was examined and, an unexpected ipsilateral deficit was evident with the chocolate distractor, unlike studies which have utilised this test in models of Parkinson’s disease.^17^ However, a difference was also evident on the contralateral side at one month, which just failed to achieve significance, and it is possible that an increase in sample size may resolve this (n = 31, Table 2).

In the modified neuroscore, an increased score was seen in the MCAO group at one and seven days; however, this resolved by one month post-MCAO. This is in line with other studies that show a high degree of recovery in neurological scores with time.^18,19^ While this test may detect deficits under certain conditions, for example in the short term after stroke, one major concern with employing tests in which there is spontaneous recovery is that, if a benefit from a therapeutic intervention is seen acutely post-MCAO, the experimenter will not know whether the treatment has truly improved functional capacity or has simply accelerated the spontaneous recovery which will naturally occur over the coming weeks. The ladder test was assessed at one and two months and did not consistently show strong deficits. While the MCAO rats made more hindpaw slips and less forepaw slips on the regular spaced rungs at one month, the overall number of foot slips was very low, indicating the rats did not struggle to cross. While a slight but non-significant increase in latency to cross the ladder was observed in both groups when the task was made increasingly difficult by spacing the rungs irregularly, the number of overall foot slips under this condition remained low.

### Counter-intuitive tests

A few tests provided results that may be considered unexpected or contrary to the initial hypothesis. On both the tapered balance beam and gait analysis, performance of the MCAO animals was significantly better at some time points compared to the Control group. For example, while the time to cross the tapered balance beam did not differ between the groups, the MCAO rats produced significantly less hindpaw slips at one month. This was surprising, as the presence of the supporting ledge is thought to alleviate the fear associated with foot slips – which might otherwise lead to a fall – and thus deficits may be exaggerated. However, this may be explained by taking into account the results of the gait analysis, which showed the MCAO rats had a reduced hindpaw base width at one month, with a trend towards the same decrease at two months. While this is also unexpected (an increased base width has been associated with MCAO at longer time points^20^), the reduced base width might mean that the rats are less likely to slip, as the hindlimbs are further away from the edge of the beam. It is possible that this decrease in base width is a compensatory mechanism together with the increase in stride length. Indeed, in addition to a reported increase in base width, other groups have reported a mild decrease in stride length using a catwalk system in rats with occlusion durations of 60 min or more.^18,20^ Thus, the duration of occlusion used in the present study may not have been sufficient to induce the magnitude of damage required to produce the same changes in parameters of gait. We also did not examine body weight as a potential confound, as was the case in the previous experiments.

Swimming behaviour also provided interesting results. The MCAO rats made slightly more corrective fore paw strokes with the impaired forelimb at one month. Though this did not achieve significance, it has been reported previously and is often attributed to disinhibition of the forepaws which are not normally used for swimming in rats.^21^ However, MCAO rats did exhibit an increase in swim speed, which on the surface might imply they are swimming better than has previously been reported in this model.^22^ The increased swim speed may be due to the recruitment of forelimbs, or increased anxiety, which has also been reported before in this model,^23^ unfortunately anxiety was not measured in these animals.

The MCAO rats also exhibited increased grip strength, although this seems unexpected, it has been reported before particularly in models of Parkinson’s disease.^24^ It is postulated that this is due to a reduction in the ability to initiate the movement to open the fore paw and release the bar, akin to dystonia.

Although, appearing counter-intuitive these tests still provide measures of dysfunction in these MCAO rats, and therefore may be of potential use if replicated in independent experiments. However, it is equally necessary to identify the underlying causes of these changes if the tests are to contribute to our understanding of the disease and potential treatments rather than simply provide further empirical measures of deficit and recovery.

### Tests in which MCAO animals did not exhibit deficits

There were a number of tests that were not sensitive to deficits and even with increased sample sizes would be unlikely to demonstrate differences between MCAO and Control groups (Table 2). One such test was amphetamine-induced rotations. Rotational bias to amphetamine has been reported in the rat MCAO model previously;^6,25^ however, these studies used longer occlusions times (60–120 min) resulting in greater damage of the dorsal and medial striatum. Amphetamine rotation has classically been associated with dopamine denervation and re-innervation of the dorsal striatum,^26^ whereas lateral lesions (the striatal area predominantly affected by MCAO) do not produce significant rotational asymmetry.^27^

Of particular interest is the fact that both the adhesive removal and cylinder tasks, which have been employed extensively in experimental stroke research, failed to reveal robust impairments at any of the tested time points. With the adhesive removal task, testing was only performed at one and two post-operative months; therefore, there is the potential that deficits may have been seen at earlier time points. Indeed, the magnitude of the somatosensory asymmetry has been shown to be larger at acute time points (1–14 days) but decreases in the chronic phase (21–30 days) using a more severe permanent proximal occlusion of the MCAO.^28^ The fact we observed a very small (but non-significant) bias in time to contact at one month, would support the notion that spontaneous recovery on this test may occur. Interestingly, the most common caveat we experienced with this test was that the rats (both Control and MCAO) ignored the stimuli and took quite a long time to contact them. This could potentially be due to the fact that the rats in this study were very well handled and did not find the administration of the sticky tape aversive. A similar effect for the cylinder task, in that at chronic time points limb use deficits were not as pronounced, has also been reported.^28^ Although, with the proximal permanent, MCAO model deficits up to 28 days have been seen in this task.^29^ With tests such as the cylinder that rely on natural exploratory behaviour, there is the potential confound that rodents lose interest with time, particularly when well habituated to the environment, and this may also have been a contributing factor.

## Limitations

Some limitations of our experiments include the choice of rat strain and the high incidence of sub-arachnoid haemorrhage. The present study used Wistar rats and these results may not transfer directly to other strains, as there are significant differences in the way different strains respond on behavioural tasks.^11^ Approximately, 20% of animals were excluded due to the lack of restoration of CBF following removal of the filament. In all cases, animals were euthanized during surgery and a sub-arachnoid haemorrhage was found on post-mortem examination. High haemorrhage rates are unfortunately not uncommon with this model. However, we would like to point out that, within our laboratory, we now use Doccol filaments (Doccol corporation, USA), which has significantly reduced this adverse event. It should also be noted that behavioural testing was carried out during the light cycle. Time of testing has been shown to have an effect on rodent performance, particularly on cognitive tasks.^30^

Despite the fact that we aimed to produce small lesions, the short occlusion time (30 min) is associated with a few limitations. For example, there was a high rate of failure of the model (15% of animals were excluded due to incomplete MCAO). While it is possible to increase the occlusion time to obtain larger infarcts, it is widely acknowledged that incomplete MCAO is a common limitation of this model and not completely dependent on occlusion duration. The smaller lesions may also explain the lack of deficits in some tests and it is possible these tests are more suitable for rodents with large, extensive infarcts.^6,25^ However, it is important to point out that the filament model has the potential to produce large lesions that could be considered to resemble malignant infarctions in humans. In this situation, rodents often receive the poorest outcome scores due to reluctance or the inability to participate in the test. It has been argued that tests that only detect deficits at acute time points, or in animals with extensive infarcts (such as neurological score) may simply be more sensitive to morbidity outcomes and not necessarily due to sensorimotor impairments. We therefore feel that obtaining smaller lesions, in which the overall impact on health was not as great a contributing factor, was an advantage to our study. Furthermore, tests that are robust enough to detect deficits in this smaller lesion model should be applicable in the inverse situation, provided the health of the animals has recovered sufficiently to participate. Finally, a large number of behavioural tests were used in this study, something that would not be recommended for routine use or in a therapeutic study. In itself, this may have acted as an intervention, enriching the rat’s environment and/or providing some degree of physical therapy, and thereby reducing the magnitude of deficits on individual tasks that would have been seen if they had been conducted alone. However, even if this was the case, and the extensive testing provided some degree of practice, compensation, neuroprotection or enhanced endogenous plasticity, the aims were still met as only tasks that are stable and robust would have been significant. Furthermore, in the clinical context, it is best practice to provide stroke patients with physical therapy, so the extensive training provided in the present experiment will further help to avoid false positives. On average, rats were given 8–15 min of testing each day, for 20–25 days in total, which is more enrichment than received in a standard laboratory environment, but it is also not considered excessive.

In conclusion, sensitive and robust tests that do not show spontaneous recovery and are performed within extended time windows are required if novel therapeutic strategies are to be successful. Here, we demonstrate a number of tests, including lateralized stepping, rotarod, apomorphine-induced rotations and the staircase task that were all able to detect long-term deficits in the relatively mild rat 30 min filament MCAO model. It is important to highlight that, even with these tests, both the variability of the model and the inherent variability seen between animals when assessing behaviour, necessitates large group sizes. To assist with this, we provide sample size calculations from the data in the present study to assist with future experimental design, including testing of therapies with an effect size of 50%.

## References

[1] Macrae IM Preclinical stroke research – advantages and disadvantages of the most common rodent models of focal ischaemia. Br J Pharmacol 2011; 164: 1062–1078. 21457227 10.1111/j.1476-5381.2011.01398.x PMC3229752

[2] DeVries AC Nelson RJ Traystman RJ Cognitive and behavioral assessment in experimental stroke research: will it prove useful? Neurosci Biobehav Rev 2001; 25: 325–42. 11445138 10.1016/s0149-7634(01)00017-3

[3] Fisher M Feuerstein G Howells DW Update of the stroke therapy academic industry roundtable preclinical recommendations. Stroke 2009; 40: 2244–2250. 19246690 10.1161/STROKEAHA.108.541128 PMC2888275

[4] Trueman RC Harrison DJ Dwyer DM A critical re-examination of the intraluminal filament mcao model: impact of external carotid artery transection. Transl Stroke Res 2011; 2: 651–661. 24323685 10.1007/s12975-011-0102-4

[5] Bancroft J Stevens A Theory and practice of histological techniques, 3rd ed Edinburgh: Churchill Livingstone, 1990.

[6] Modo M Stroemer RP Tang E Neurological sequelae and long-term behavioural assessment of rats with transient middle cerebral artery occlusion. J Neurosci Methods 2000; 104: 99–109. 11163416 10.1016/s0165-0270(00)00329-0

[7] Klein A Sacrey L-AR Whishaw IQ The use of rodent skilled reaching as a translational model for investigating brain damage and disease. Neurosci Biobehav Rev 2012; 36: 1030–1042. 22227413 10.1016/j.neubiorev.2011.12.010

[8] Bruce-Keller AJ Umberger G McFall R Food restriction reduces brain damage and improves behavioral outcome following excitotoxic and metabolic insults. Ann Neurol 1999; 45: 8–15. 9894871

[9] Roberge M-C Messier C Staines WA Food restriction induces long-lasting recovery of spatial memory deficits following global ischemia in delayed matching and non-matching-to-sample radial arm maze tasks. Neuroscience 2008; 156: 11–29. 18672030 10.1016/j.neuroscience.2008.05.062

[10] Chen J Sanberg PR Li Y Intravenous administration of human umbilical cord blood reduces behavioral deficits after stroke in rats. Stroke 2001; 32: 2682–2688. 11692034 10.1161/hs1101.098367

[11] Kunze A Zierath D Drogomiretskiy O Variation in behavioral deficits and patterns of recovery after stroke among different rat strains. Transl Stroke Res 2014; 5: 569–576. 24711015 10.1007/s12975-014-0337-y

[12] Monville C Torres EM Dunnett SB Comparison of incremental and accelerating protocols of the rotarod test for the assessment of motor deficits in the 6-OHDA model. J Neurosci Methods 2006; 158: 219–223. 16837051 10.1016/j.jneumeth.2006.06.001

[13] Dowd E Monville C Torres EM The corridor task: a simple test of lateralised response selection sensitive to unilateral dopamine deafferentation and graft-derived dopamine replacement in the striatum. Brain Res Bull 2005; 68: 24–30. 16325001 10.1016/j.brainresbull.2005.08.009

[14] Tornero D Wattananit S Grønning MM Human induced pluripotent stem cell-derived cortical neurons integrate in stroke-injured cortex and improve functional recovery. Brain 2013; 136: 3561–77. 24148272 10.1093/brain/awt278

[15] Olsson M Nikkhah G Bentlage C Forelimb akinesia in the rat Parkinson model: differential effects of dopamine agonists and nigral transplants as assessed by a new stepping test. J Neurosci 1995; 15: 3863–3875. 7751951 10.1523/JNEUROSCI.15-05-03863.1995 PMC6578238

[16] Hall S Schallert T Striatal dopamine and the interface between orienting and ingestive functions. Physiol Behav 1988; 44: 469–471. 3237839 10.1016/0031-9384(88)90307-1

[17] Schallert T Hall S “Disengage” sensorimotor deficit following apparent recovery from unilateral dopamine depletion. Behav Brain Res. 1988; 30: 15–24. 3139011 10.1016/0166-4328(88)90003-4

[18] Encarnacion A Horie N Keren-Gill H Long-term behavioral assessment of function in an experimental model for ischemic stroke. J Neurosci Methods 2011; 196: 247–257. 21256866 10.1016/j.jneumeth.2011.01.010 PMC3539723

[19] Farr TD Carswell HVO Gsell W Estrogen receptor beta agonist diarylpropiolnitrile (DPN) does not mediate neuroprotection in a rat model of permanent focal ischemia. Brain Res 2007; 1185: 275–282. 17942083 10.1016/j.brainres.2007.09.009

[20] Parkkinen S Ortega FJ Kuptsova K Gait impairment in a rat model of focal cerebral ischemia. Stroke Res Treat 2013; 2013: 410972. 23533959 10.1155/2013/410972 PMC3603709

[21] Stoltz S Humm JL Schallert T Cortical injury impairs contralateral forelimb immobility during swimming: a simple test for loss of inhibitory motor control. Behav Brain Res 1999; 106: 127–132. 10595428 10.1016/s0166-4328(99)00100-x

[22] Bingham D Martin SJ Macrae IM Watermaze performance after middle cerebral artery occlusion in the rat: the role of sensorimotor versus memory impairments. J Cereb Blood Flow Metab 2012; 32: 989–999. 22373646 10.1038/jcbfm.2012.16 PMC3367220

[23] Quinn LP Grundy RI Campbell CA A novel behavioural registration system LABORAS and the social interaction paradigm detect long-term functional deficits following middle cerebral artery occlusion in the rat. Brain Res 2005; 1031: 118–124. 15621019 10.1016/j.brainres.2004.10.036

[24] Dunnett SB Torres EM Annett LE A lateralised grip strength test to evaluate unilateral nigrostriatal lesions in rats. Neurosci Lett 1998; 246: 1–4. 9622193 10.1016/s0304-3940(98)00194-3

[25] Hudzik TJ Borrelli A Bialobok P Long-term functional end points following middle cerebral artery occlusion in the rat. Pharmacol Biochem Behav 2000; 65: 553–562. 10683498 10.1016/s0091-3057(99)00243-9

[26] Dunnett SB Björklund A Schmidt RH Intracerebral grafting of neuronal cell suspensions. IV. Behavioural recovery in rats with unilateral 6-OHDA lesions following implantation of nigral cell suspensions in different forebrain sites. *Acta Physiol Scand Suppl* 1983; 522: 29–37. 6426250

[27] Fricker RA Annett LE Torres EM The placement of a striatal ibotenic acid lesion affects skilled forelimb use and the direction of drug-induced rotation. Brain Res Bull 1996; 41: 409–416. 8973847 10.1016/s0361-9230(96)00083-4

[28] Schallert T Fleming SM Leasure JL CNS plasticity and assessment of forelimb sensorimotor outcome in unilateral rat models of stroke, cortical ablation, parkinsonism and spinal cord injury. Neuropharmacology 2000; 39: 777–787. 10699444 10.1016/s0028-3908(00)00005-8

[29] Farr TD Carswell HVO Gallagher L 17beta-Estradiol treatment following permanent focal ischemia does not influence recovery of sensorimotor function. Neurobiol Dis 2006; 23: 552–562. 16759876 10.1016/j.nbd.2006.04.009

[30] Winocur G Hasher L Aging and time-of-day effects on cognition in rats. Behav Neurosci 1999; 113: 991–997. 10571481 10.1037//0735-7044.113.5.991

